# Disentangling Action from Social Space: Tool-Use Differently Shapes the Space around Us

**DOI:** 10.1371/journal.pone.0154247

**Published:** 2016-05-04

**Authors:** Ivan Patané, Tina Iachini, Alessandro Farnè, Francesca Frassinetti

**Affiliations:** 1 Department of Psychology, University of Bologna, Bologna, Italy; 2 ImpAct Team, Lyon Neuroscience Research Centre, INSERM U1028, CNRS UMR5292, Lyon, France; 3 UCBL, Lyon I University, Lyon, France; 4 Laboratory of Cognitive Science and Immersive Virtual Reality, Department of Psychology, Second University of Naples, Caserta, Italy; 5 Hospices Civiles de Lyon, Neuro-immersion & Mouvement and Handicap, Lyon, France; 6 Fondazione Salvatore Maugeri, Clinica del Lavoro e della Riabilitazione, IRCCS–Istituto Scientifico di Castel Goffredo, Mantua, Italy; Centre de Neuroscience Cognitive, FRANCE

## Abstract

Converging evidence suggests close relationships between the action and social space representations. The concepts of peripersonal space, as defined by cognitive neuroscience, and interpersonal space, as defined by social psychology, refer to approximately the same spatial area surrounding our bodies. The aim of this study was thus to assess experimentally whether the peripersonal (PPS) and interpersonal space (IPS) represent a similar psychological entity. Were this true, they should share some functional features. Here we tested tool-use dependent plasticity, known to modulate PPS, but still unexplored in the IPS. Results from two experiments converge in showing that tool-use remapped the action-related PPS, measured by a Reaching-distance toward a confederate, but did not affect the social-related IPS, measured by a Comfort-distance task. These findings indicate that PPS and IPS rely on dissociable plastic mechanisms and suggest that, at least in the present experimental conditions, there is no full functional overlap between these two spatial representations.

## Introduction

The representation of the space around the body has attracted the ever-growing interest of scholars from cognitive neuroscience to social psychology [1]. In their seminal monkey study, Rizzolatti and colleagues [2,3] defined the “peripersonal space” (PPS) as the sector of space coded by neurons responding to both tactile and visual stimuli near the body. Since then, many authors refer to the PPS as an action space that offers a multisensory interface for body–objects interactions [4,5] somewhat overlapping the reaching space [6–9]. Yet, in our environment we interact not only with inanimate objects, but also with other people. Typically, humans maintain a distance around their bodies, called “interpersonal space” (IPS), any intrusion into which by others may cause discomfort [10,11]. Both PPS and IPS are plastic: for instance, PPS is ‘lengthened’ by tool use [12–14], whereas IPS can be modified by emotional and socially relevant interactions [15,16].

In this regard, it has been demonstrated that social interactions between confederates can also modulate the plastic boundary of PPS [17]. After an economic interaction, for example, the participant’s PPS boundary extended to include the partner at a distance, but only if the confederate behaved cooperatively. In fact, merely knowing that the seen partner acts upon events near one's body has been shown to influence the visual-tactile integration in the PPS [18]. This “social” influence was thus interpreted as due to a top-down modulation on the sensorimotor representations of PPS. Accordingly, more complex social information, such as perceived morality of another person, can exert a top-down influence on PPS regulation [19].

In the light of this recent evidence showing that PPS is sensitive to some social factors, the question has been raised as to whether PPS and IPS share some functional features [20]. The idea is emerging that functional links, or maybe even functional identity, may exist between the action-related and the social-related space. Our rationale for testing this hypothesis is that, were this true, any sensory-motor plastic change induced in the PPS representation should concurrently modulate the IPS one. To this aim, here we applied a tool-use paradigm, known to modify both near-body space perception and arm length representation [21–25], to assess whether PPS and IPS are similarly affected, or not. In order to compare tool-use effects onto the action- and social-related spatial representations, we adopted two classical measures taken as index of the extent, or “size”, of the PPS and the IPS, respectively. On the one hand, the reachability judgement task was here borrowed from the neuro-cognitive domain to measure the PPS [7,25]. In this task, participants have to estimate whether stimuli presented at various distances are reachable or not by extending their limb (without moving), thereby stressing the sensorimotor and potential action aspects of spatial perception. Since in this study we focused on individual-to-individual relationships, we applied a modified version of the reachability task, by asking participants to estimate the reachability distance towards an unfamiliar person. On the other hand, the comfort-distance task, drawn from proxemics studies of social psychology, was adopted to measure the IPS. In this task, participants have to stop the confederate at the point where they still feel comfortable with the other’s proximity [15,26], stressing thus the social aspect of spatial perception. Importantly, participants performed the two tasks during both Passive and Active approach conditions. In the former case, the confederate walked across the room towards the participant, whereas in the latter case, the participant walked towards the confederate. Given that PPS and IPS seem to share a similar size in the Active, but not in the Passive approach [19,20], we asked whether plastic effects of tool-use on spatial processing are modulated by the active or passive way of interacting with the environment.

## Experiment 1

### Materials and method

#### Ethics Statement

Participants gave written consent to take part in the study, which was carried out in accordance with the 2008 Helsinki Declaration and approved by the Ethics Committee of the University of Bologna.

#### Participants

Twenty-four participants (9 females, age range = 19–27 years, mean age = 22 years), naive to the experimental hypothesis, volunteered for the study. Participants had no self-reported history of neurological or psychiatric diseases, and were right-handed as assessed by administration of the Edinburgh Handedness Inventory [27]. We calculated our target sample size using an estimated effect size, f, of 0.2, which would require a sample of 24 participants for the study to be powered at 80%. The a priori established sample size was also used as data-collection stopping rule, i.e. when 24 volunteers were administered with the tasks in a fully counterbalanced design, no more participant was recruited for this study.

#### Procedure

**Fig 1:**
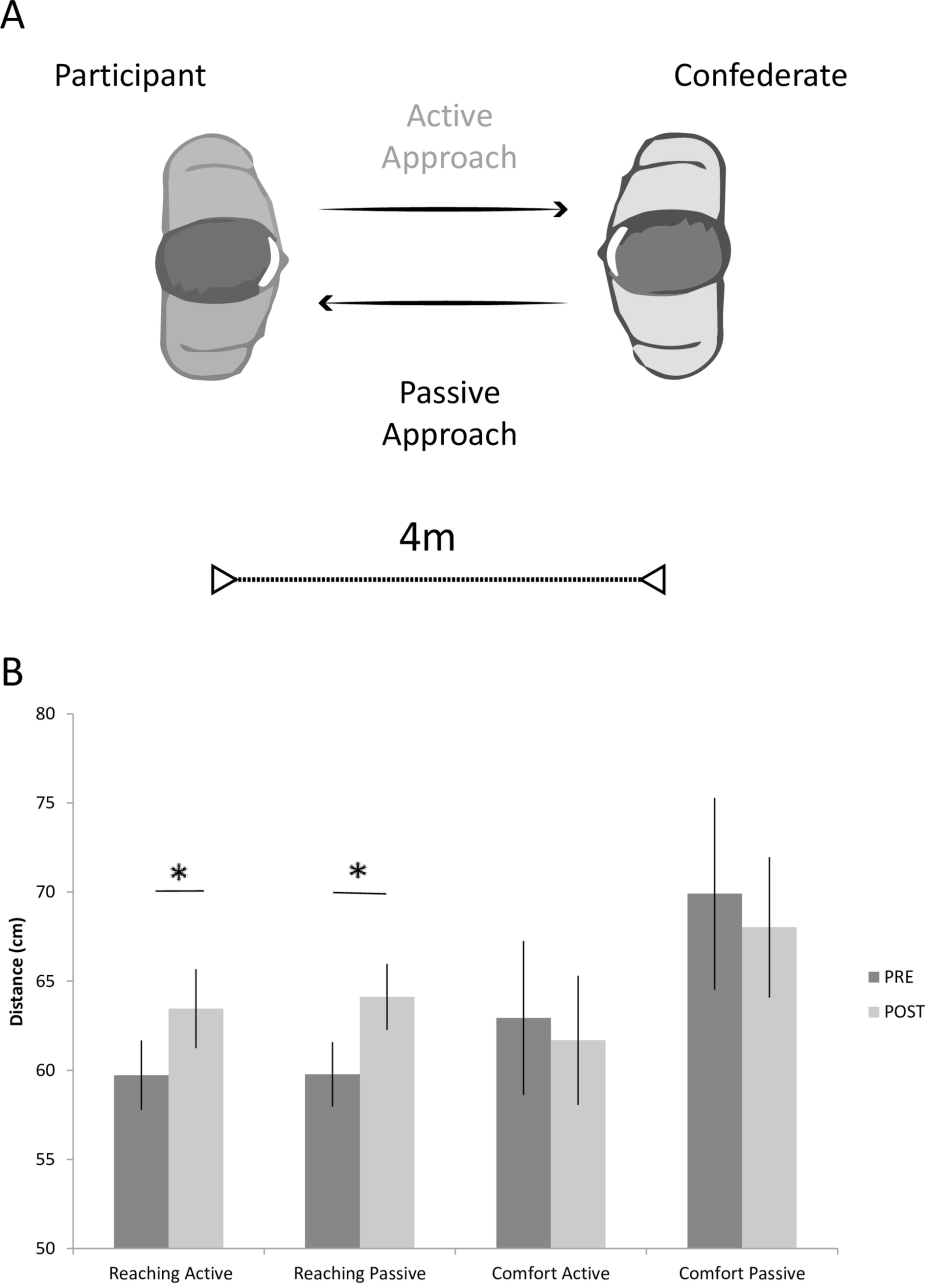
Experimental procedure and results of Experiment 1. Participants performed Reaching-distance and Comfort-distance tasks in the Active and Passive approach conditions (A). Participants were told to stop a confederate (Passive condition) or themselves (Active condition) when they could either reach the confederate, in the Reaching-distance, or feel comfortable with their interpersonal distance, in the Comfort-distance task. The graph (B) shows the average group distance (in cm) as a function of task, condition, and session. Error bars indicate standard errors of the mean. Asterisks indicate significant differences in Reaching-distance before (PRE, dark grey) and after (POST, light grey) tool-use (two-tailed *t* tests *p* ≤ .002).

All participants were tested in the same room (7.5 x 6.5 m) by one experimenter and two confederates. The experimental protocol consisted of two tasks (Reaching- and Comfort-distance, Fig 1A), designed to respectively measure the PPS and the IPS, that were performed in both Passive and Active approach conditions. Testing began with a participant positioned at a fixed location in the room and one confederate standing at a distance of four meters (starting position). In the Active conditions, participants approached a confederate and had to comply with either of two instructions: “stop yourself at the distance you think you can reach the confederate” (Active Reaching-distance), or “stop yourself at the shortest distance you feel comfortable with" (Active Comfort-distance). In the Passive conditions, the confederate approached the participant who had to either: “stop the confederate at the distance you think you can reach the confederate” (Passive Reaching-distance) or “stop the confederate at the shortest distance you feel comfortable with" (Passive Comfort-distance). The distance between the participant and the confederate was recorded with a digital laser measurer (Agatec, model DM100, error ±.003 m), as the distance from the confederate’s to the participant’s sternum.

This procedure was repeated twice in separate blocks of five trials per each condition: before and after 15 minutes of tool-use. Two different male confederates, unknown to participants, were involved in pre and post-tool sessions. One of the confederates approached and was approached by the participant for the entire duration of the first session before tool-use, whereas the second confederate was introduced in the experimental session after tool-use. Moreover, to avoid any confounds due to aesthetical or idiosyncratic features of the two confederates, the order of the confederate facing the participant in the pre and post tool-use session was also counterbalanced between participants. The confederates had to wear the same neutral casual clothes and to maintain a neutral expression and same speed in walking throughout the experimental session. Confederates’ gaze was kept looking straight ahead at participants’ eye-level. Before starting the experiment all participants received an explanation of the task and had four practice trials with the experimenter. Then the first confederate was introduced.

During tool-use, participants stood still in front of a table and were required to use a 70 cm-long rake to reach and to retrieve, one at a time, a total of 36 tokens, located out of the reaching space, at a distance of ≈85 cm from the participants’ sternum. Tokens were randomly positioned by the experimenter at different azimuthal and radial locations in order to cover the whole participant's action space. Participants were instructed to make a continuous, fluid movement to retrieve the targets and to move them close to their body midline. There was no time constraint and participants were required to be as accurate as possible.

Immediately after 15 minutes of tool-use, participants were asked to perform the two tasks (Comfort-distance and Reaching-distance) in both the Active and Passive approach conditions. Throughout the experimental sessions before and after too-use, the participants stood with their arms extended along their trunk and were instructed to close their eyes between each trial. The order of the tasks was counterbalanced between subjects.

### Data analysis

To test whether tool-use may ‘extend’ either the Reaching-distance or the Comfort-distance measures or both, the mean distances obtained in the different experimental conditions were compared through a three-way ANOVA with Session (Pre-Post tool-use), Task (Reaching-Comfort distance), and Approach (Passive-Active approach) as within-participant factors. Newman-Keuls post-hoc test was used to analyse significant effects. Partial eta squared (η^2^_p_) and Cohen’s *d* were used to report effect size. Additional variables included as covariates in exploratory analyses were age, sex, handedness and years of formal education. Since inclusion of these covariates in the analyses did not modify the results, they were left out of the final analyses.

### Results

Statistical analysis revealed a significant effect of the Approach variable (F_1,23_ = 6.84, p = .015, η^2^_*p*_ = 0.23), due to overall participant-confederate distance being larger in the Passive approach than Active approach condition. More interestingly, the significant Task x Session interaction (F_1,23 =_ 6.25, p = .020, η^2^_*p*_ = 0.21) showed that the Reaching-distance (p = .018), but not the Comfort-distance (p = .333), was significantly extended after tool-use as compared to before. The Reaching-distance before tool-use was the shortest distance as compared to all other conditions (all ps≤.018). The ANOVA also found a statistically significant interaction between Approach and Task variables (F_1,23_ = 16.08, p < .001, η^2^_*p*_ = 0.41). In the Passive approach, the Comfort-distance was larger than the Reaching-distance (p < .001), whereas in the Active approach, Comfort and Reaching distances did not significantly differ (p = .797). The Passive-Comfort condition was significantly larger as compared to all other conditions (all ps < .001). Significant main effects and interactions are reported in Table 1.

**Table 1:** Significant main effects and interactions: F test, p value, partial eta squared, mean ±S.E.M. in cm.

**Approach**	**Active**	**Passive**
(F_1,23_ = 6.84, p = .015, η^2^_*p*_ = 0.23)	61.95 ±2.72	65.45 ±2.87
**Session x Task**	**Pre Reaching**	**Post Reaching**
(F_1, 23_ = 6.25, p = .020, η^2^_*p*_ = 0.21)	59.75±1.80	63.79 ±1.94
	**Pre Comfort**	**Post Comfort**
	66.42 ±4.72	64.85 ± 3.65
**Task x Approach**	**Active Reaching**	**Active Comfort**
(F_1,23_ = 16.08, p < .001, η^2^_*p*_ = 0.41)	61.59±2.02	62.31±3.89
	**Passive Reaching**	**Passive Comfort**
	61.95±1.75	68.96 ±4.46

Since the three-way interaction was not significant (F_1,23_< 1, p = .635, η^2^_*p*_ = 0.01), the effect of tool-use in the Reaching-distance task was present both in the Active (two tailed *t*(23) = 3.35, p = .002, d = 0.71) and in the Passive condition (two tailed *t*(23) = 4.12, p < .001, d = 0.83), but was not evident in the Comfort-distance task for either approach (p≥.475, Fig 1B). Furthermore, in line with previous studies, at baseline in the Active approach no significant difference between Comfort and Reaching-distance appeared (two tailed *t*(23) < 1, p = .388, d = 0.18). Overall, these results reveal that tool-use modifies the reaching space, but not the comfort space.

### Discussion

The issue addressed in this study is whether the PPS and the IPS underlie two different functional representations of the space that closely surrounds our body. We therefore investigated changes of PPS and IPS after tool-use by measuring the spatial distance between the participant and another person in a Reaching- and a Comfort-distance task. Two main results emerged from the first experiment: first, we replicated previous findings, indicating PPS and IPS seem to refer in some circumstances to a similarly sized sector of space. Indeed, Reaching- and Comfort-distances had a similar extent when participants walked toward stimuli (Active approach). By contrast, the Comfort-distance was larger than the Reaching-distance when participants stood still while participants were approached (Passive approach) [19,20]. Second, more interestingly, the result of the first experiment goes beyond previous findings by revealing that the tool-use manipulation was effective in shaping selectively the perceived reaching space (PPS), both during the Active and the Passive approaches, but not the perceived comfort space (IPS).

When considering this dissociation, it is worth noticing that during tool-use session the rake allowed participants to reach targets located beyond the natural reaching distance of their hand, and thereby, the rake significantly extended their action space. In line with previous works on tool-use [12–14,22], it is thus likely that an extension of the sensorimotor representation of the arm length was critical to determine such an extension of action space. However, a possible alternative interpretation may be that the mere act of reaching repetitively for objects placed in the space in front of the participant, regardless of the length of the used tool, may have affected the estimated reaching distance. In order to control for this potential alternative explanation, we conducted another study (Experiment 2), where as a control, participants used a short rake that did not offer effective extension of their arm length [14,21,23,25]. In Experiment 2, we anticipated that only using a tool that elongates the arm length would modulate the PPS, as measured by the Reaching-distance task, whereas using a short tool should be ineffective.

Concerning the lack of a significant effect of tool-use on the comfort space estimation, one could argue that participants may have used non-spatial (e.g., temporal) strategies, since the two confederates were positioned at a constant distance from the participant prior to and following tool-use session. Thus, to avoid this potential confound in Experiment 2 confederates were positioned at one of three different initial distances from participants.

## Experiment 2

By analogy with previous work estimating reachable distance from objects [25], Experiment 2 tested the hypothesis that tools that increase the natural range of action, as compared to those which do not, may “extend” the reachable space estimate toward another individual. To this aim, participants performed the same tasks as in the previous experiment, but they wielded either a long or a short tool. Since in Experiment 1 the tool-use effect was observed in both Passive and Active approaches, and the Reaching- and Comfort-distances were similar only in the Active one, in Experiment 2 we focused on the Active approach. Furthermore, the confederate was randomly located at three different positions, thus preventing participants from using a non-spatial strategy to stop themselves in front of the confederate. We predicted an increased distance in the estimated reaching space after use of the long, but not the short tool. Finally, we should expect no change in the estimated comfort space after either long or short tool session, even after preventing the use of non-spatial strategies.

### Materials and method

#### Ethics Statement

All participants volunteered and provided written informed consent. The study was approved by the Ethics Committee of the University of Bologna and was performed in accordance with the ethical standards laid down in the Declaration of Helsinki.

#### Participants

Forty-eight healthy volunteers (24 females, age range = 18–27, mean age = 22.47 years) naive to the purpose of the study participated in this experiment. None of these participants took part in the previous experiment. All participants had normal or corrected-to-normal vision, no history of neurological or psychiatric diseases, and all but two were right-handed, as assessed by the Edinburgh Handedness Inventory [27].

#### Procedure

The experimental setting and procedure were similar to those of Experiment 1, with the following exceptions. Participants were randomly assigned to one of two groups: Long tool group or Short tool group. In the former group, the rake was the same as in the previous experiment (70 cm-long). In the latter group, the tool was a 10 cm-long weight-matched rake. The distance at which target objects were placed during the short tool-use session was adapted at around ≈25 cm from the participant’s body. Participants performed the Reaching- and Comfort-distance tasks in separate blocks, under Active approach only, prior to and following the tool-use session. As in Experiment 1, each participant faced a different confederate before and after the tool-use sessions. In each condition the position of the confederate, and thereby the initial distance from participant, could be either 3.5 m, 4 m, or 4.5 m from the participant. Each different confederate position was randomly administrated four times, yielding a total of twelve trials per each condition.

### Data analysis

Statistical analyses consisted on a three-way ANOVA taking the variables Session (Pre-Post tool-use) and Task (Reaching-Comfort distance) as within-participant factors and the variable Tool group (Short-Long tool group) as between-participant factors. Partial eta squared and Cohen’s *d* are used to report effect size. Significant sources of variance were explored by Newman–Keuls post-hoc tests. In addition, because preliminary analyses of variance failed to reveal any significant effect of the different confederate positions (i.e., neither main effect of Confederate distance nor any interaction with the other terms reached statistical significance, all *F*s≥ 1.19, all *p*s≥ .313), this factor is not considered further here.

### Results

**Fig 2:**
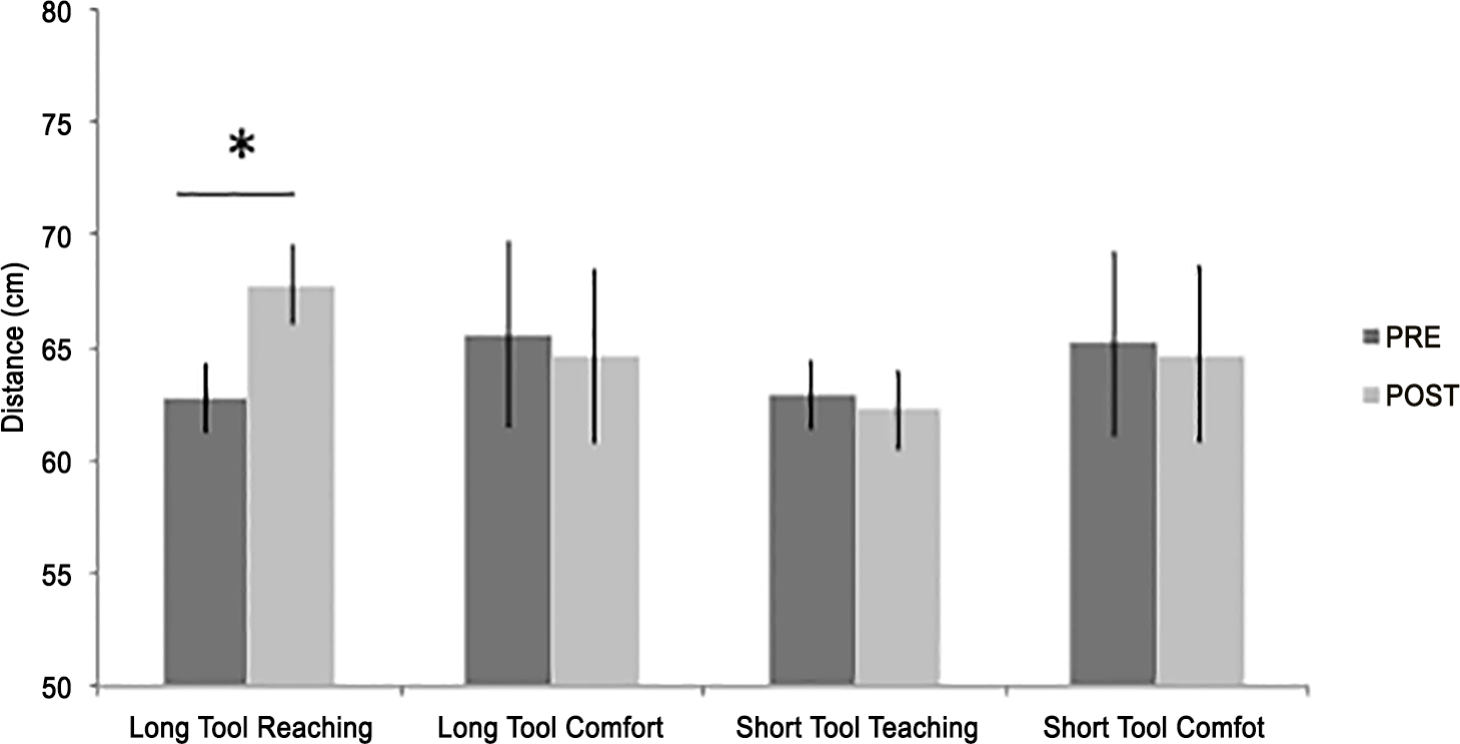
Results of Experiment 2. The graph shows the average distance (in cm) as a function of group, task, and session. Error bars indicate standard errors of the mean. Asterisk indicates a significant difference between Reaching-distance before (PRE, dark grey) and after (POST, light grey) long tool-use session.

The ANOVA revealed a significant Task x Session interaction (F_1,46_ = 6.64, p = .013, η^2^_*p*_ = 0.13), explained by the fact that the pre-tool Reaching-distance showed the shortest amplitudes as compared to the post-tool Reaching-distance (p = .024) and to the Comfort distance before (p = .012) as well as after tool-use (p = .024). Crucial to the present investigation, a significant Task x Session x Tool group interaction emerged (F_1,46_ = 7.25, p = .010, η^2^_*p*_ = 0.14). Post hoc tests revealed that the interaction was driven by an increased Reaching-distance estimation in the Long tool group following the use of long tool with respect to before (p = .001), whilst no significant difference between before and after long tool-use amplitudes was found in the Comfort-distance task (p = .839). On the other hand, in the Short tool group post hoc tests showed no significant differences prior to vs. following the use of short tool in either the Reaching- (p = .836) or the Comfort-distance task (p = .652, Fig 2). In addition, none of the other terms in the ANOVA reached statistical significance (all *F*s ≥ 2.62). In sum, the critical statistical interaction Task x Session x Tool group witnesses that only the use of a long, but not a short tool affected the reachable space estimation. In sharp contrast, neither the use of a short nor of a long tool modulated the comfort space estimation.

### Discussion

Experiment 2 replicates previous findings in showing the tool-use-dependent plasticity of PPS (e.g., [13,24]). In particular, only after using the 70 cm-long tool that extends the arm reachable space the PPS was extended, as measured by the Reaching-distance task. As predicted, and also in line with previous studies (e.g., [14,25]), using a shorter tool that provides no functional extension to the arm did not influence the PPS. As far as the question of whether the IPS representation may be plastically shaped by the use of tools, the second experiment confirms the lack of effect of tool-use on the Comfort-distance task.

## General Discussion

This study brings two major new findings. As first important result, tool-use can modify the reachability estimate toward another person, a potentially relevant social stimulus. At odds with previous tool-use studies, by focusing on individual-to-individual spatial relationships, i.e., the interpersonal distance [28], we provide here, to the best of our knowledge, the first demonstration that tool-use affects the estimated reaching-distance between two conspecifics.

So far, previous research has demonstrated that the extent of PPS representation is dynamically shaped by use of tools that increase the arm length [12,23,24,29,30], as using short tool, which does not extend action potentiality, is not sufficient to “elongate” the PPS [14,21,25,31]. Noteworthy, most if not all studies on PPS have so far tested participants while facing objects in absence of conspecifics, although people around us often represent the most behaviorally relevant stimuli in everyday life. In this respect, the findings from the present work indicate that extending the arm action capability also affects the perceived reaching distance from another individual.

The second and most important result is that tool-use modulates the PPS, as measured by the Reaching-distance task, but not the social IPS, as measured by the Comfort-distance task. This dissociation is particularly interesting as the estimated reaching and comfort space had similar size before tool-use, specifically in the Active approach, that is when participants moved toward the confederate (for similar findings see also [20]). In light of such a similarity, it might appear surprising that tool-use does not influence the social space surrounding the body, but only the space representation for action. Before considering the theoretical implications of this finding, consistently observed across two experiments, we wish to emphasize that our design was aimed at controlling for potentially confounding variables, such as stimulus familiarity and non-spatial response biases, that could account for the lack of effect of tool use on the social space representation. Several pieces of evidence from social psychological studies indicate that repeated contact and familiarity with individuals affect interpersonal distances (e.g., [32,33]). In order to avoid this possible confound, here participants were not exposed to the same person in the session prior to and following tool-use: confederates’ identity differed between pre- and post-tool use sessions. In addition, since in the second experiment the confederate was not located at the same initial distance from the participant, it is unlikely that the internally replicated finding concerning the Comfort-distance task may be due to any response/strategy bias. In Experiment 2, indeed, participants could not use non-spatial cues, such as the time spent while walking or the number of walking steps.

Thus, the results of both experiments converge in indicating that social and action space representations do not fully share the same functional properties. Moreover, but fundamental to the present study, this tool-use dependent dissociation actually fits well some anatomo-functional differences between PPS and IPS. In this regard, to identify specific areas associated with IPS a previous study [34] focused on which brain regions respond to interpersonal space intrusion by social stimuli (i.e., conspecifics) versus non-social stimuli (i.e., objects). To this end, Holt and colleagues used both an out-of-scanner behavioral task, similar to that we used here involving actual intrusion of space by a conspecific, and a within-scanner virtual task designed to simulate interpersonal space intrusion through the apparent approach or withdrawal of a conspecific’s face vs. the same approaching/receding movement of inanimate objects. They reported the dorsal intraparietal sulcus and ventral premotor cortex to be more sensitive to approaching than to receding faces. Contrary to what one could expect if the same neuronal populations would code for IPS and PPS, this differential activity did not emerge when contrasting approaching versus receding objects (i.e., non-social objects that are typically effective stimuli for eliciting PPS neuron responses in the monkey and humans). Additionally, the authors demonstrated that the strength of the functional connectivity between these parietal-frontal regions was negatively correlated with participants’ interpersonal distance, measured behaviorally outside of the scanner. These results suggest a link between areas responding to virtual approaching faces in the scanner and individual preferences in interpersonal distance. On the other hand, specific areas associated with PPS have been identified by electrophysiological approaches in primates, which have well-established that neuronal populations coding for visual stimuli near the body are located in interconnected regions involving the inferior parietal lobe, intraparietal sulcus, premotor cortex, and putamen [35,36,2]. Similarly, evidence from humans indicates a representation of PPS in the putative homologous parietal and frontal regions [37–39]. Indeed fMRI adaptation studies, allowing to isolate functionally selective neuronal populations, demonstrated that some neuronal populations within the premotor and parietal cortices (inferior parietal lobe and anterior intraparietal sulcus), as well as the putamen are involved in the coding for approaching, non-socially relevant objects [39–41]. Taken together, these findings suggest that, when considering the social vs. non-social nature of nearby stimuli, the PPS and IPS representations seem not completely overlapping also at the neural level.

To summarise, researchers from cognitive psychology, neuroscience and social psychology have recently looked with great interest at the possibility that the same underlying processes mediate interactions with both inanimate and animate objects within the space surrounding our body [26]. Although some previous studies have highlighted a communality between peri- and inter-personal spatial representations, here we disclose a functional dissociation between the two sectors of the space at the behavioural level. Whilst it has been shown that PPS plasticity may be sensitive to top-down influences coming from social experiences with the individual facing us [17–20], here we report that the sensorimotor plasticity induced by tool-use on the action space does not influence the social space between individuals. Critically, this finding was observed while concurrently demonstrating that the very same tool-use manipulation was effective in extending the PPS also when measured from a more social perspective, namely by asking to estimate the reachable distance with respect to a person, instead of an object. Therefore, the results reported here provide novel insights to the current debate, spanning from cognitive neuroscience to social psychology [9,20,28,34,39,42], about some critical functional features of action and social space representations, notably their plasticity.

We propose that, to devise appropriate models of PPS and IPS, one should bear in mind that, at least in the present experimental conditions, there is no complete functional overlap between these sectors of space. We believe that a single example of functional dissociation is indeed sufficient to warn scholars to refrain from risky conflations between the two concepts, and critically inform current theoretical models about space perception. After all, even if we could use one single space representation as frame-of-reference for interacting with other objects and people, it would make sense that the spatial representations that we use flexibly conform to the task at hand.
